# Radial contrast enhancement on brain magnetic resonance imaging diagnostic of primary angiitis of the central nervous system: a case report and review of the literature

**DOI:** 10.1186/1752-1947-8-26

**Published:** 2014-01-27

**Authors:** Kartheek Ganta, Aisha Mohsin Malik, James B Wood, Michael C Levin

**Affiliations:** 1 Department of Neurology, University of Tennessee Health Science Center, Memphis, TN, USA; 2 Department of Neurology, Veterans Administration Medical Center, Memphis, TN, USA; 3 Department of Radiology Services, Veterans Administration Medical Center, Memphis, TN, USA; 4 Department of Radiology, University of Tennessee Health Science Center, Memphis, TN, USA

**Keywords:** Brain MRI, Central nervous system, Gadolinium enhancement, Vasculitis

## Abstract

**Introduction:**

Primary angiitis of the central nervous system is a rare disease of unclear etiology. There is no single test diagnostic of primary angiitis of the central nervous system. We report an unusual pattern on brain magnetic resonance imaging that might be specific for primary angiitis of the central nervous system.

**Case presentation:**

A 47-year-old Caucasian man developed progressive bilateral hand tremor, difficulty walking, cognitive slowing and headache. A physical examination showed bilateral hand tremor with dysmetria, hyperreflexia and abnormal gait. Magnetic resonance imaging of his brain showed bilateral, symmetrical, increased intensity on T2-weighted images concurrent with linear contrast enhancement in a radial distribution throughout his white matter, sparing subcortical regions in his centrum semiovale, corona radiata, basal ganglia and brainstem. Magnetic resonance spectroscopy demonstrated elevated choline and decreased N-acetyl aspartate. Except for elevated protein and lymphocytic pleocytosis, examination of his cerebrospinal fluid showed no abnormalities. Serological tests for rheumatologic, vasculitic, paraneoplastic, infectious and peroxisomal disorders were negative. A brain biopsy revealed primary angiitis of the central nervous system. Our patient was treated with steroids and intravenous cyclophosphamide, with improvement in signs and symptoms as well as changes on magnetic resonance imaging.

**Conclusion:**

Bilateral, symmetrical, increased intensity on T2-weighted images concurrent with linear contrast enhancement in a radial distribution throughout the white matter on magnetic resonance imaging of the brain should be recognized as a feature of primary angiitis of the central nervous system, and might avoid the need for a brain biopsy to diagnose primary angiitis of the central nervous system.

## Introduction

Primary angiitis of the central nervous system (PACNS) can be defined as vasculitis affecting exclusively the central nervous system (CNS) without systemic disease. It is a rare disease of unclear etiology with an estimated incidence of 2.4 per 1,000,000 [1]. It manifests clinically with headache, altered mentation and a variety of focal neurological deficits [2]. There is no specific test to diagnose PACNS. Magnetic resonance imaging (MRI) of the brain typically reveals multifocal white and gray matter signal abnormalities. Less commonly, leptomeningeal enhancement may occur [2]. Magnetic resonance angiography is not useful in PACNS because it cannot demonstrate vasculitic changes in blood vessels smaller than the major intracranial arteries or their primary branches. Cerebral angiography can demonstrate ectasia and stenosis, however sensitivity is only 60% [3,4]. Secondary causes of vasculitis need to be ruled out by thorough blood and cerebrospinal fluid (CSF) testing. A brain biopsy is still the gold standard for diagnosis of PACNS. Brain biopsies in PACNS can have a false negative rate of up to 25% [3]. Brain biopsy is associated with a transient and permanent morbidity of 14% and 4% respectively [5].

We present the case of a patient with biopsy-proven PACNS in which the MRI abnormalities displayed a radial distribution of contrast enhancement, a rarely reported pattern that likely corresponds to inflamed cerebral vessels and perivascular regions.

## Case presentation

A 47-year-old Caucasian man initially presented with a two-month history of progressive bilateral hand tremor, difficulty walking, behavioral changes and headache. On examination he was afebrile, with poor memory recall, bilateral action and postural tremor, diffuse hyperreflexia with the presence of Babinski signs bilaterally, frontal release signs, bilateral dysmetria and marked truncal ataxia resulting in an inability to walk. His medical history included hypertension, anxiety disorder and tobacco use. Medications included citalopram and lisinopril.

**Figure 1:**
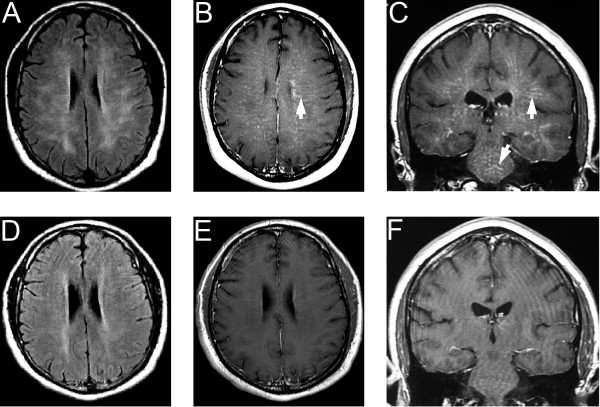
**Brain magnetic resonance images before (A-C) and after (D-F) treatment. (A)** T2-weighted FLAIR imaging demonstrates bilateral, diffuse hyperintense lesions in the white matter with sparing of the cortical U fibers. T1-weighted images following intravenous gadolinium administration in the **(B)** axial and **(C)** coronal planes show bilateral, symmetrical, linear contrast enhancement in a radial distribution throughout the white matter of the cortex and brainstem (arrows). **(D)** Following treatment with steroids and cyclophosphamide, T2-weighted FLAIR imaging demonstrates a dramatic improvement of the hyperintense abnormalities and almost complete resolution of gadolinium enhancement on the T1-weighted images in the **(E)** axial and **(F)** coronal planes.

T2-weighted fluid attenuated inversion recovery (FLAIR) brain MRI revealed diffuse, symmetrical, increased intensity throughout the white matter in his centrum semiovale and corona radiata (Figure 1A) concurrent with bilateral linear enhancement in a radial distribution on the gadolinium-contrasted study (Figure 1B,C). The subcortical ‘U’ fibers of his white and grey matter were spared. No abnormalities were noted in his cerebellum and corpus callosum. Magnetic resonance spectroscopy (MRS) demonstrated elevated choline, decreased N-acetyl aspartate and no evidence of lactate (not shown). MRI scans of his cervical and thoracic spine were normal. Magnetic resonance and computed tomography angiograms were normal.

Basic and specialized laboratory tests were either normal or negative. Specifically, these included a complete blood count; comprehensive metabolic profile; erythrocyte sedimentation rate; C-reactive protein; vitamins B12, D, E and folate; thyroid profile; human immunodeficiency virus; hepatitis A, B and C panel; angiotensin-converting enzyme; serum protein electrophoresis; serum galactocerebrosidase; arylsulfatase; very long chain fatty acids; polymerase chain reaction for Whipple’s disease; mycoplasma immunoglobulin (Ig) G and IgM; Lyme IgG and IgM; blood cultures; and rheumatologic studies (anti-nuclear antibody, rheumatoid factor, C3/C4, anti-Ro, anti-La, anti-double-stranded deoxyribonucleic acid (DNA), anti-ribonucleoprotein, c/p-anti-neutrophil cytoplasmic antibody, anti-SS-A/B, anti-myeloperoxidase). A urine analysis, urine drug screen and chest X-ray were all negative.

A lumbar puncture showed an opening pressure of 26cm H_2_O (normal: 50 to 180mm H_2_O), 240 white blood cells per mm^3^ (89% lymphocytes, 11% monocytes), 10 red blood cells per mm^3^, protein 196mg/dL (normal: 15 to 45mg/dL), and glucose 49mg/dL (normal: 45 to 85mg/dL). Cerebrospinal fluid (CSF) bacterial, viral, fungal, acid-fast bacilli stains and cultures were negative, as was a multiple sclerosis panel (oligoclonal bands, IgG index, and quantitative IgG). His CSF was also negative for anti-Yo and anti-Hu antibodies and cytology was negative for malignancy. CSF polymerase chain reaction studies were negative for herpes simplex viruses 1 and 2, varicella zoster virus, John Cunningham (JC) virus, Epstein-Barr virus, cytomegalovirus and parvovirus.

Our patient was initially treated with intravenous antibiotics until infectious studies came back negative. He was then started on intravenous dexamethasone followed by a tapering dose of oral prednisone. Our patient showed a dramatic clinical improvement, was ambulatory in two days, and was discharged from the hospital. One month later, while on oral steroids, he was clinically stable. A repeat lumbar puncture showed an opening pressure of 18cm H_2_O, 0 white cells per mm^3^, protein 106mg/dL, and glucose 52mg/dL. T2-weighted FLAIR MRI of his brain showed decreased intensity of the white matter abnormalities with persistence of subtle bands of contrast enhancement (not shown). Nerve conduction studies and electromyography showed no evidence of neuropathy.

Three months later, our patient’s gait began to deteriorate. An examination revealed increased tremulousness, dysmetria and spastic gait. T2-weighted FLAIR MRI of his brain revealed worsening intensity of the white matter changes (not shown). An MRI of his spine showed extensive, diffuse, continuous T2 white matter hyperintensities in his cervical and thoracic spinal cord (C7 to T9, not shown). His CSF revealed 73 white cells per mm^3^ (98% lymphocytes, 2% monocytes), protein 141mg/dL and glucose 93mg/dL. His IgG level was elevated at 9.9mg/dL but oligoclonal bands were absent.

**Figure 2:**
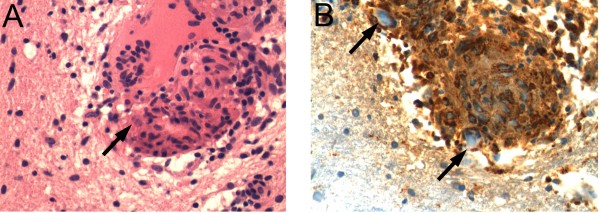
**Pathology of the brain biopsy. (A)** Hematoxylin and eosin staining shows the development of a non-caseating granuloma with giant cells (arrow). **(B)** Staining with anti-CD68 antibodies demonstrates the presence of macrophages within a developing granuloma with giant cells (arrow).

A right frontal brain biopsy showed numerous perivascular non-caseating granulomas consistent with the granulomatous pattern of PACNS (Figure 2). Our patient was treated with oral prednisone and monthly intravenous cyclophosphamide (750mg/m^2^) for 12 months. There was improvement in his headache and balance. A neurological examination showed decreased tremor and dysmetria, and his gait had almost normalized. Follow-up brain and spine MRI scans showed improvement in the white matter hyperintensities on FLAIR imaging (Figure 1D) as well as on the gadolinium-enhanced images (Figure 1E,F).

## Discussion

MRI abnormalities are present in over 95% of cases of PACNS confirmed by histology [6,7], although they are reportedly non-specific. The most common findings are focal signal abnormalities with characteristics of multiple bilateral cerebral infarcts involving gray and deep white matter [8,9]. Leptomeningeal enhancement may be associated with parenchymal lesions [2] or be an isolated MRI finding [10]. Less commonly, signal abnormalities have been reported to involve only the deep white matter [8,11]. Hemorrhagic lesions may also be present [8,12].

MRI of our patient’s brain revealed radial linear enhancements on the gadolinium-contrasted study associated with hyperintensities throughout his white matter on FLAIR imaging. MRS in our patient demonstrated elevated choline and decreased N-acetyl aspartate, indicating a breach in neuronal membranes and neuronal loss, respectively.

We searched PubMed for relevant published articles (up to May 2013), including electronic early release publications. Search terms included: primary angiitis/vasculitis of the CNS, granulomatous angiitis, vasculitis/angiitis, MRI granulomatous vasculitis, lymphocytic vasculitis, and lymphocytic angiitis. Relevant articles were retrieved and prioritized for inclusion and their references were checked for additional material when appropriate. After a thorough PubMed search, we found four cases in the literature (Table 1) that included similar MRI findings in biopsy-proven PACNS [13-15]. These MRI findings may indicate severe but reversible perivascular inflammation causing blood–brain barrier disruption and injury of surrounding white matter. The radial distribution of these enhancing signal abnormalities follows the path of blood vessels, which we believe may be a specific marker for PACNS. This configuration is not seen in the MRI scans of patients with multiple sclerosis or post-viral demyelination, which show ring-like, lobular or fusiform signal abnormalities. Notably, MRS was not reported in any of these cases. MRS findings were described in few case reports but were found to be non-specific and not in agreement with each other [16-19].

**Table 1:** Magnetic resonance imaging and clinical findings of biopsy-proven cases of primary angiitis of the central nervous system

**Reference**	**Age (y)/sex**	**Symptoms**	**MRI findings**	**Location of abnormalities**	**MRI with gadolinium contrast description**	**Biopsy**
Shoemaker *et al.*[13]	45/M	Dysesthesias, loss of sensation, gait imbalance	T2 focal areas of high signal	Brainstem, cerebellum, cerebral white matter	Multiple focal areas of enhancement, some were linear	Small arteries infiltrated by lymphocytes, macrophages, neutrophils
Campi *et al*. [14]	50/M	Progressive severe paraparesis	Small punctate T2 hyperintensities	Subcortical, supra- and infratentorial white matter	Punctate contrast enhancement	Inflammation of small vessels with lymphocytes and granulocytes
Campi *et al*. [14]	29/F	Headache, diplopia, ataxia	Small high signal foci on T2 images	Supratentorial white matter	Enhancement of Virchow-Robin spaces	Vasculitis of small parenchymal vessels, fibrinoid necrosis
Hassan *et al*. [15]	38/F	Tremor, gait ataxia, incoherent mentation	T2 white matter hyperintensities	Diffuse	Contrast enhancement in a linear radiating fashion	Perivascular inflammation with T cells, B cells and macrophages
Patient in this report	47/M	Tremor, headache, gait imbalance	T2 with diffuse hyperintensity	Centrum semiovale and corona radiata	Bilateral linear enhancements in a radiating fashion	Perivascular non-caseating granulomas

F, female; M, male; MRI, magnetic resonance imaging.

We believe that the presence of the brain MRI findings reported in our patient should prompt physicians to consider a diagnosis of PACNS, in which case aggressive and timely treatment may result in dramatic improvements in neurologic function, reduce long-term neurologic disability and be life saving. This report is only one a few cases, but we now see a potential pattern of diagnostic importance.

## Conclusion

The presence of bilateral, symmetrical, increased intensity on T2-weighted images concurrent with linear contrast enhancement in a radial distribution throughout the white matter on brain MRI may be a diagnostic signature of PACNS. If more such cases are reported, brain biopsy and its associated morbidity and mortality could potentially be avoided.

## Consent

Written informed consent was obtained from the patient for publication of this case report and accompanying images. A copy of the written consent is available for review by the Editor-in-Chief of this journal.

## Abbreviations

CNS: central nervous system; CSF: cerebrospinal fluid; FLAIR: fluid attenuated inversion recovery; Ig: immunoglobulin; MRI: magnetic resonance imaging; MRS: magnetic resonance spectroscopy; PACNS: primary angiitis of the central nervous system.

## Competing interests

The authors declare that they have no competing interests.

## Authors’ contributions

KG and AMM analyzed and interpreted the patient data regarding the clinical presentation and were major contributors in writing the manuscript. JBW performed and interpreted the MRI data. MCL reviewed all of the data and made major contributions to writing and editing the manuscript. All authors read and approved the final manuscript.

## References

[B1] Salvarani C Brown RD Jr Calamia KT Christianson TJ Weigand SD Miller DV Giannini C Meschia JF Huston J 3rd Hunder GG Primary central nervous system vasculitis: analysis of 101 patients Ann Neurol 2007 62 5 442 451 10.1002/ana.21226 17924545

[B2] Harris KG Tran DD Sickels WJ Cornell SH Yuh WT Diagnosing intracranial vasculitis: the roles of MR and angiography AJNR 1994 15 317 330 8192080 PMC8334625

[B3] Calabrese LH Furlan AJ Gragg LA Ropos TJ Primary angiitis of the central nervous system: diagnostic criteria and clinical approach Cleve Clin J Med 1992 59 3 293 306 10.3949/ccjm.59.3.293 1516217

[B4] Vollmer TL Guarnaccia J Harrington W Pacia SV Petroff OA Idiopathic granulomatous angiitis of central nervous system Arch Neurol 1993 50 9 925 930 10.1001/archneur.1993.00540090032007 8363446

[B5] Woodworth GF McGirt MJ Samdani A Garonzik I Olivi A Weingart JD Frameless image-guided stereotactic brain biopsy procedure: diagnostic yield, surgical morbidity, and comparison with the frame-based technique J Neurosurg 2006 104 2 233 237 10.3171/jns.2006.104.2.233 16509497

[B6] Calabrese LH Duna GF Lie JT Vasculitis in central nervous system Arthritis Rheum 1997 40 7 1189 1201 9214418 10.1002/1529-0131(199707)40:7<1189::AID-ART2>3.0.CO;2-4

[B7] Stone JH Pomper MG Roubenoff R Miller TJ Hellmann DB Sensitivities of noninvasive tests for central nervous system vasculitis: a comparison of lumbar puncture, computed tomography, and magnetic resonance imaging J Rheumatol 1994 21 177 182 7966069

[B8] Greenan TJ Grossman RI Goldberg HI Cerebral vasculitis: MR imaging and angiographic correlation Radiology 1992 182 65 72 1727311 10.1148/radiology.182.1.1727311

[B9] Pierot L Chiras J Debussche-Depriester C Dormont D Bories J Intracerebral stenosing arteriopathies: contribution of three radiological techniques to the diagnosis J Neuroradiol 1991 18 32 48 1880560

[B10] Negishi C Sze G Vasculitis presenting as primary leptomeningeal enhancement with minimal parenchymal findings AJNR 1993 14 26 28 8427102 PMC8334474

[B11] Finelli PF Onyiuke HC Uphoff DF Idiopathic granulomatous angiitis of the CNS manifesting as diffuse white matter disease Neurology 1997 49 1696 1699 10.1212/WNL.49.6.1696 9409370

[B12] Hellmann DB Roubenoff R Healy RA Wang H Central nervous system angiography: safety and predictors of a positive result in 125 consecutive patients evaluated for possible vasculitis J Rheumatol 1992 19 568 572 1593579

[B13] Shoemaker EI Lin ZS Rae-Grant AD Little B Primary angiitis of the central nervous system: unusual MR appearance AJNR 1994 15 331 334 8192081 PMC8334615

[B14] Campi A Benndorf G Filippi M Reganati P Martnelli V Terreni MR Primary angiitis of the central nervous system: serial MRI of brain and spinal cord Neuroradiology 2001 43 599 607 10.1007/s002340100561 11548164

[B15] Hassan AS Trobe JD McKeever PE Gebarski SS Linear magnetic resonance enhancement and optic neuropathy in primary angiitis of the central nervous system J Neuroophthalmol 2003 23 2 127 131 10.1097/00041327-200306000-00004 12782924

[B16] Yu XL Liu AF MA L Yan CZ Zhao YY Shan PY Primary angiitis of the central nervous system: a case report Chin Med J (Engl) 2011 124 17 2782 2785 22040443

[B17] Lee Y Kim JH Kim E Park SH Yim YJ Sohn CH Chang KH Tumor-mimicking primary angiitis of the central nervous system: initial and follow-up MR features Neuroradiology 2009 51 10 651 659 10.1007/s00234-009-0546-3 19529928

[B18] Panchal NJ Niku S Imbesi SG Lymphocytic vasculitis mimicking aggressive multifocal cerebral neoplasm: MR imaging and MR spectroscopic appearance AJNR Am J Neuroradiol 2005 26 642 645 15760879 PMC7976487

[B19] Beppu T Inoue T Nishimoto H Nakamura S Nakazato Y Ogasawara K Ogawa A Primary granulomatous angiitis of the central nervous system: findings of magnetic resonance spectroscopy and fractional anisotropy in diffusion tensor imaging prior to surgery: case report J Neurosurg 2007 107 873 877 10.3171/JNS-07/10/0873 17937238

